# Regulation of Serotonin 1A Receptor SUMOylation by SENP2 and PIASxα

**DOI:** 10.3390/ijms222413176

**Published:** 2021-12-07

**Authors:** Sugandha Gupta, Mengya Wang, Yoshiaki Azuma, Nancy A. Muma

**Affiliations:** 1 Department of Pharmacology and Toxicology, School of Pharmacy, University of Kansas, Lawrence, KS 66045, USA; sugandha4894@ku.edu (S.G.); m375w089@ku.edu (M.W.); 2 Department of Molecular Bioscience, College of Arts and Science, University of Kansas, Lawrence, KS 66045, USA; azumay@ku.edu

**Keywords:** SUMOylation, PIAS, SENP, estrogen, estradiol, serotonin 1A receptors

## Abstract

Serotonin 1A receptors (5-HT1ARs) are implicated in the control of mood, cognition, and memory and in various neuropsychiatric disorders such as depression and anxiety. As such, understanding the regulation of 5-HT1ARs will inform the development of better treatment approaches. We previously demonstrated 5-HT1ARs are SUMOylated by SUMO1 in the rat brain. Agonist stimulation increased SUMOylation and was further enhanced when combined with 17β-estradiol-3-benzoate (EB), which are treatments that cause the transient and prolonged desensitization of 5-HT1AR signaling, respectively. In the current study, we identified the protein inhibitor of activated STAT (PIAS)xα as the enzyme that facilitates SUMOylation, and SENP2 as the protein that catalyzes the deSUMOylation of 5-HT1ARs. We demonstrated that PIASxα significantly increased in the membrane fraction of rats co-treated with EB and an agonist, compared to either the EB-treated or vehicle-treated groups. The acute treatment with an agonist alone shifted the location of SENP2 from the membrane to the cytoplasmic fraction, but it has little effect on PIASxα. Hence, two separate mechanisms regulate SUMOylation and the activity of 5-HT1ARs by an agonist and EB. The effects of EB on 5-HT1AR SUMOylation and signaling may be related to the higher incidence of mood disorders in women during times with large fluctuations in estrogens. Targeting the SUMOylation of 5-HT1ARs could have important clinical relevance for the therapy for several neuropsychiatric disorders in which 5-HT1ARs are implicated.

## 1. Introduction

Serotonin (5-HT) and 5-HT1A receptor (5-HT1AR) signaling plays important roles in the pathology and treatment of depression, anxiety, and related mood disorders [1]. For example, mice constitutively lacking 5-HT1ARs are more anxious [2] and 5-HT1AR agonists are used as anxiolytic drugs. Furthermore, the desensitization (reduced responsiveness) of 5-HT1ARs is essential for the therapeutic effects of antidepressants [3,4]. Depression affects 1 in 6 Americans, but only 2/3 of those patients are effectively treated with current oral medication approaches [5]. Women are almost twice as likely to suffer from depression as men, and fluctuations in estrogens contribute to depression and other mood disorders [6]. Estrogens can regulate 5-HT1AR signaling [7]. Our previous study demonstrated that the estrogen 17β-estradiol benzoate (EB) causes a partial desensitization of 5-HT1AR signaling [8] and the combination of the antidepressant fluoxetine and EB produces a robust and rapid desensitization [9]. However, the mechanisms of this regulation are unclear.

Understanding the mechanisms underlying the regulation of 5-HT1ARs will inform the development of more effective therapeutic approaches. We previously demonstrated that 5-HT1ARs are SUMOylated by SUMO1 (SUMO1-5-HT1ARs) in the rat brain [10]. SUMOylation is a post-translational modification whereby a small ubiquitin-like modifier protein (SUMO) is covalently conjugated to a target protein in a manner similar to ubiquitination. SUMOylation can alter protein function, protein to protein interactions, and protein localization [11]. We demonstrated that the agonist stimulation of 5-HT1ARs increases this SUMOylation and EB further enhances the SUMOylation of 5-HT1ARs. Using discontinuous gradient centrifugation and receptor binding assays with (+)8-hydroxy-2-dipropylaminotetralin (8-OH-DPAT), a 5-HT1A/7R agonist, we found that SUMO1-5-HT1ARs have a low capacity for agonist binding [10]. Furthermore, using discontinuous gradient centrifugation and Western blotting, SUMO1-5-HT1ARs did not co-localize with Gαz, a Gα to which the receptor couples [10,12]. Taken together, these results suggest SUMO1-5-HT1ARs are not active receptors and that SUMOylation contributes to the desensitization of 5-HT1ARs caused by treatment with 8-OH-DPAT and EB.

SUMO proteins are conjugated to their substrates via an isopeptide bond between the di-glycines at the C-terminus of SUMO and a lysine residue in the substrate protein. In mammals, the process of SUMOylation occurs through a series of enzymatic reactions analogous to ubiquitination [11,13]. SUMOylation does not occur in vivo for most substrates without SUMO E3 ligases, which facilitate the transfer of the SUMO group to the target protein. The protein inhibitor of activated STAT (PIAS) family proteins has E3 ligase activity and mammalian cells contain four members of the PIAS family, PIAS1, PIAS3, and PIASy, as well as splice variants PIASxα and PIASxβ [11,14]. Different PIAS proteins have preferences for different substrates and have selectivity for different SUMO proteins. E3 ligase activity has been demonstrated for several other proteins, such as RanBP2, Polycomb protein 2, and histone deacetylase 4, but a very limited number of substrates have been identified as E3 ligases to date.

DeSUMOylation is catalyzed primarily by sentrin-specific proteases known as SENPs [15]. There are six mammalian SENPs that are involved in deSUMOylation, SENPs 1–3 and 5–7. SENPs act not only to deconjugate the SUMO moiety from substrate proteins, but also act in the maturation of SUMO proteins by exposing the C-terminal diglycine moiety on SUMO proteins to initiate the SUMOylation process. Different SENPs have different activities in deconjugation and maturation processing, as well as substrate specificity. SENP1 displays little specificity for the deconjugation of SUMO2 and SUMO3, while it is essential for the deconjugation of SUMO1-modified proteins [16,17]. SENP2 catalyzes deconjugation better than maturation processing and deconjugates SUMO-1, SUMO-2 and SUMO-3 from SUMOylated Ran GTPase-activating protein 1 with similar efficiency. In contrast, SENP2 possesses different substrate specificities during processing; for example, it processes SUMO2 more efficiently than SUMO1 and SUMO3 [11]. SENP3 and SENP5 show both maturation processing and deconjugating activities for SUMO2 and SUMO3 but possess neither for SUMO1. SENP6 and SENP7 have deconjugating activities for poly SUMO2/3 chains, while exerting very weak processing and deconjugating activity on monomeric SUMO modified substrates [11].

The goal of this study was to determine the mechanisms involved in the regulation of the SUMOylation of 5-HT1ARs. To address this question, we first investigated which PIAS and SENP proteins regulate the SUMOylation of 5-HT1ARs. Then we used two drug treatment strategies shown to increase the SUMOylation of 5-HT1ARs, agonist stimulation using 8-OH-DPAT and the combination of EB and 8-OH-DPAT, which further increased SUMOylation. A previous study suggested that local concentrations of the PIAS E3 ligases play a role in determining which E3 ligase catalyzes the SUMOylation of a target protein [18]. Furthermore, the study demonstrated that the translocation of the E3 ligase to the substrate plays a role in regulating the SUMOylation of the substrate. Based on these observations, we hypothesized that the PIAS protein(s) catalyzing the SUMOylation of 5-HT1ARs would be expressed in the cell membrane and drug treatments that increased the SUMOylation of 5-HT1AR would increase the membrane localization of the PIAS protein. To test this hypothesis, we ectopically expressed different PIAS proteins in mouse neuroblastoma 2a (N2a) cells to determine their effects on SUMO1-5-HT1ARs and measured the levels of PIAS proteins following the treatment of rats with 8-OH-DPAT or a co-treatment of EB and 8-OH-DPAT. Additionally, a decrease in SENP proteins in the membrane could result in less deSUMOylation, thereby increasing the SUMOylation of 5-HT1ARs. Therefore, we over-expressed SENPs1, 2, and 6 to determine which SENP deSUMOylates 5-HT1ARs and then measured the levels of SENP2 following drug treatment.

We found that overexpression of PIASxα increased SUMOylation and SENP2 decreased SUMOylation of 5-HT1ARs in N2a cells. The treatment of rats with the 5-HT1A/7R agonist 8-OH-DPAT decreased SENP2 proteins in the membrane. In contrast, the combined treatment with 8-OH-DPAT and EB on rats increased PIASxα proteins in the membrane. These results provide a further understanding of the mechanisms by which agonists and estrogens regulate the SUMOylation of 5-HT1ARs and thereby the sensitivity of 5-HT1AR signaling.

## 2. Results

### 2.1. The Overexpression of PIAS Proteins on the SUMOylation of 5-HT1ARs

**Figure 1:**
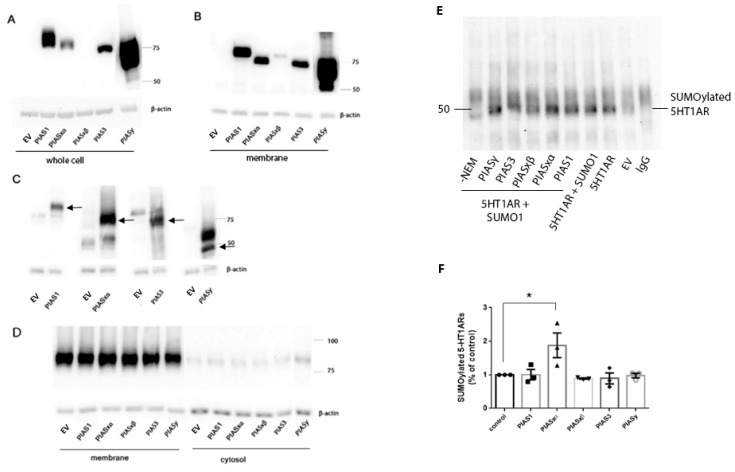
The expression of PIAS proteins in N2a cells. N2a cells were transfected with flag-tagged PIAS plasmid constructs. Immunoblots were prepared with the whole cell lysates (**A**) and the membrane fraction of cells (**B**) and incubated with anti-flag antibody. The lanes loaded with cells transfected with PIASy were separated from those for the other PIAS proteins due the high level of expression of this construct and the need to avoid interference. Because of this issue, the image for the PIASy lane is separated from the other lanes in the image although examined on the same blot. Based on the amino acid sequence, PIASy is approximately 56 kD but can be both SUMOylated and ubiquitinated, suggesting that the higher molecular weight band on the immunoblots may be a post-translationally modified PIASy protein band. (**C**) The expression of each PIAS protein was detected using specific anti-PIAS antibodies in the membrane fraction from cells transfected with empty vector (EV) and cells transfected with plasmids for each PIAS protein. (**D**) The separation of membrane fractions was verified by immunoblotting using Na^+^/K^+^ ATPase as plasma membrane marker. (**E**) The effect of PIAS proteins on SUMOylation of 5-HT1ARs. N2a cells were transfected with plasmids that express 5-HT1AR, SUMO1 and a PIAS protein as indicated. Various amounts of empty vector were added equalize the amount of plasmid constructs transfected in total per plate. 5-HT1ARs were SUMOylated by SUMO1 in N2a cells as shown by immunoprecipitation with a SUMO1 antibody and western blotting with an antibody for 5-HT1AR. IgG indicates immunoprecipitation with the same amount of mouse IgG instead of a mouse anti-SUMO1 antibody using lysate from cells transfected with both SUMO1 and 5-HT1AR as a negative control. EV indicates cells were transfected with empty vector. Cell lysates were also harvested in the absence of NEM (-NEM) as indicated which would result in the deSUMOylation of proteins since NEM inhibits sentrin proteases. (**F**) Data presented in the graph are from three separate experiments and are the mean ± SEM as well as indicating individual data points. One-way ANOVA shows there is significant effect of transfection of PIAS on SUMOylation of 5-HT1ARs (F(5,12) = 4.612, *p* = 0.0140). Dunnett’s multiple comparisons post-hoc test shows SUMOylation of 5-HT1AR significantly increased in the group transfected with 5-HT1AR, SUMO1, and PIASxα compared to the group transfected with the 5-HT1AR and SUMO1, * indicates (*p* < 0.05).

To investigate which PIAS proteins are involved in the SUMOylation of 5-HT1ARs, we transfected flag-tagged PIAS constructs into N2a cells. The transfections of the PIAS constructs were verified by immunoblotting with an anti-flag antibody and all PIAS proteins appeared at the appropriate molecular weight on the immunoblots. Surprisingly, there were large variations in the expression of the PIAS proteins in the whole cell lysate, despite the fact that the PIAS proteins were all expressed using the same mammalian expression vector under the control of a CMV promotor. The PIASy construct expressed most abundantly followed by the PIAS1 construct. The expression levels of transfected PIAS3 constructs were less abundant than the PIAS1 construct. Transfected PIASxα protein levels were low compared to the other PIAS proteins and the expression of PIASxβ was so low in the whole cell lysate that PIASxβ was barely detected (Figure 1A).

It is important to know the relative levels of expression of the PIAS proteins after transfections because differences in the subsequent SUMOylation levels could be the result of higher expression levels of one PIAS protein over another. Since there were large variations in the expression of our PIAS constructs in the whole cell lysates following transfection, we examined the levels of PIAS proteins in the membrane fractions where the SUMOylated 5-HT1AR exist. Similar to the PIAS protein expression in the whole cell lysate, the expression of transfected PIASy in the membrane fraction was by far the most abundant, followed by PIAS1 and PIAS3. PIASxα and PIASxβ protein levels were higher relative to the other PIAS proteins in the membrane fraction than in the whole cell lysate. PIASxα protein levels were similar to PIAS3 protein levels in the membrane fraction, whereas they were much lower in than PIAS3 in the whole cell lysate. The higher relative levels of membrane-associated PIASxα and PIASxβ compared to that in the whole cell lysate (Figure 1A,B) suggests that PIASxα and PIASxβ may traffic to cell membranes. The expression level of endogenous and transfected PIAS proteins in the membrane fraction were detected using specific anti-PIAS antibodies. Results show N2a cells express endogenous PIAS proteins but at relatively low levels (Figure 1C). The expression of PIAS proteins in the cell membrane was higher in the cells transfected with PIAS constructs than in the cells transfected with an empty vector, demonstrating both successful transfection and the expression of the PIAS proteins and the endogenous levels of each PIAS protein in the membrane fraction of cells (Figure 1C). Na^+^, K^+^ ATPase, and a plasma membrane marker was used to verify the separation of membrane and cytosolic fractions (Figure 1D). The results show abundant Na^+^, K^+^, and ATPase in the membrane fraction, but little in the cytosolic fraction, indicating a successful separation of the membrane and cytosol fractions.

The SUMOylation of 5-HT1ARs was examined in N2a cells using immunoprecipitation and immunoblotting. N2a cells were co-transfected with 5-HT1ARs and SUMO1. SUMO1 modified proteins were isolated from cell lysates by immunoprecipitation with a mouse monoclonal anti-SUMO1 antibody and were examined on immunoblots with a rabbit polyclonal anti-5-HT1AR antibody to detect SUMOylation of both endogenous and transfected 5-HT1AR. We observed bands around 55 kDa (Figure 1E), the predicted molecular size for the SUMO1–5-HT1AR complex as previously detected in different regions of the rat brain [10]. To further verify that the 55 kDa band on the immunoblot was SUMO-5-HT1AR, we harvested cells in buffer made without NEM, an irreversible cysteine peptidase inhibitor that inhibits the sentrin proteases responsible for reversing SUMOylation, thereby preventing the deSUMOylation of proteins. Samples prepared without NEM showed dramatic reductions in SUMO1–5-HT1AR protein band intensity, thus confirming that the band at around 55 kDa represents the SUMO1–5-HT1ARs.

Next, we examined the effect of different PIAS proteins on the SUMOylation of 5-HT1ARs. N2a cells were co-transfected with 5-HT1AR and SUMO1 and either constructs expressing PIAS proteins or an empty vector. SUMO1 proteins were immunoprecipitated and examined on immunoblots with a rabbit polyclonal anti-5-HT1AR antibody. A one-way ANOVA showed the significant effect of transfection (F(5,12) = 4.612, *p* = 0.014). Dunnett’s multiple comparisons post-hoc test showed that PIASxα significantly increased the SUMOylated 5-HT1ARs compared to the cells transfected with 5-HT1ARs and SUMO1 (Figure 1E,F). Importantly, although PIAS1, PIAS3, and PIASy constructs were abundantly expressed and present in the membrane (Figure 1A,B) these PIAS proteins did not alter the SUMOylation of 5-HT1ARs.

### 2.2. The Effect of EB and 8-OH-DPAT on PIAS Proteins in Rat PVN

8-OH-DPAT increases the SUMOylation of 5-HT1ARs and E2 potentiates the 8-OH-DPAT-induced increase in the SUMOylation of 5-HT1A receptors in the hypothalamus of ovariectomized female rats (Li and Muma, 2013). To begin to determine the mechanisms underlying the effect of the agonist and EB treatment on the SUMOylation of 5-HT1ARs, we examined the expression levels of different PIAS proteins in the membrane fraction of the PVN of the hypothalamus of rats following treatment with 8-OH-DPAT and EB. Specific anti-PIAS1, anti-PIASxα, anti-PIAS3, and anti-PIAS4 antibodies were used to detect the expression level of corresponding PIAS proteins.

**Figure 2:**
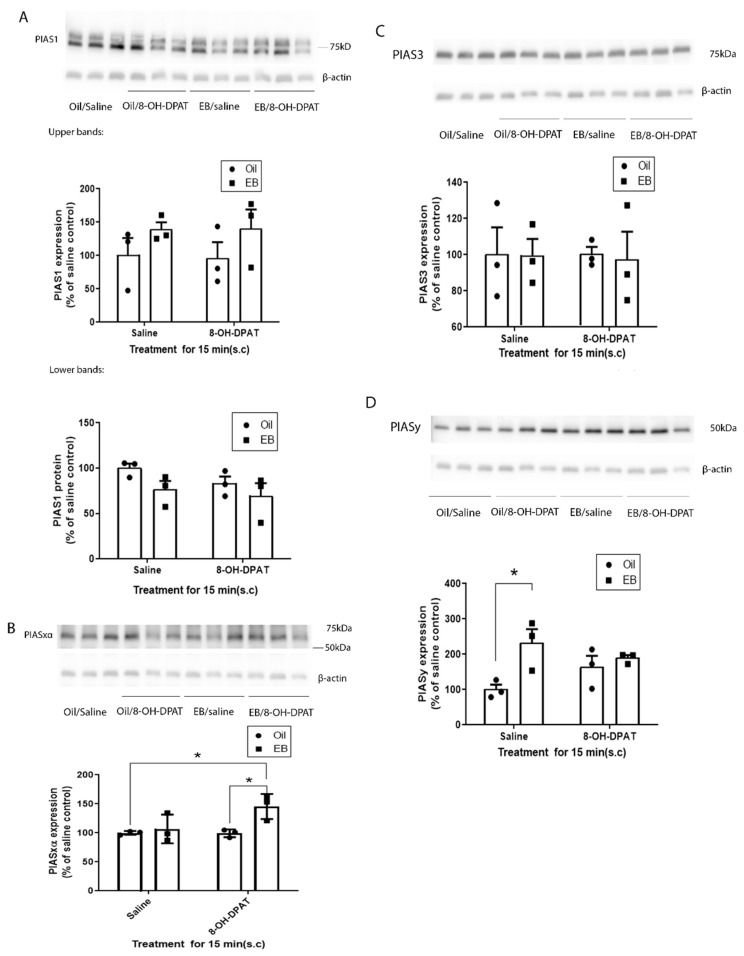
The effect of EB and 8-OH-DPAT on the PIAS proteins in the rat PVN. Ovariectomized female rats were treated with10 μg/kg EB or sesame oil once daily for 2 days and 20 h later treated with 200 μ/kg 8-OH-DPAT or saline for 5 min before sacrifice. Three rats per group were analyzed on western blots to determine the effect of EB and 8-OH-DPAT on the PIAS1 (**A**), PIASxα (**B**), PIAS3 (**C**) and PIASy (**D**). In (**B**), two-way ANOVA shows there is significant effect of EB on the expression of PIASxα. Tukey’s multiple comparison test shows treatment of EB and 8-OH-DPAT together significantly increased PIASxα in the PVN membrane fraction compared to the group treated with vehicle and 8-OH-DPAT alone * (*p* < 0.05). In (**D**), two-way ANOVA shows there is significant effect of EB on the expression of PIASy. Tukey’s multiple comparisons post-hoc test shows PIASy was increased significantly in the group treated with EB alone compared to the vehicle-treated group * (*p* < 0.05).

For PIAS1, two bands around its predicted molecular weight were observed on immunoblots (Figure 2A). Both bands were measured to quantify the effect of treatments on the expression of PIAS1. A two-way ANOVA showed no significant effect of EB or 8-OH-DPAT treatment or interactions on the expression level of PIAS1 (upper bands, two-way ANOVA, main effect of EB: F(1,8) = 3.041, *p* = 0.119; main effect of 8-OH-DPAT treatment: F(1,8) = 0.0066, *p* = 0.937; interaction between EB and 8-OH-DPAT treatment: F(1,8) = 0.01447, *p* = 0.907; lower bands, two-way ANOVA, main effect of EB: F(1,8) = 3.55, *p* = 0.096; main effect of 8-OH-DPAT treatment: F(1,8) = 1.50, *p* = 0.256; interaction between EB treatment and 8-OH-DPAT treatment: F(1,8) = 0.250, *p* = 0.630).

For PIASxα expression levels, a two-way ANOVA demonstrated a significant effect of EB treatment but there was no significant effect of 8-OH-DPAT or the interaction between EB and 8-OH-DPAT (two-way ANOVA, main effect of treatment EB: F(1,8) = 7.45, *p* = 0.026; main effect of 8-OH-DPAT treatment: F(1,8) = 3.82, *p* = 0.086; interaction between EB treatment and 8-OH-DPAT treatment: F(1,8) = 4.086, *p* = 0.078). Tukey’s multiple comparisons post-hoc test showed rats treated with EB followed by 8-OH-DPAT showed a significant increase, of about 40%, in the PIASxα level in the rat PVN membrane fraction compared to the 8-OH-DPAT-treated and vehicle-treated rats (Figure 2B). The expression of PIASxα was unchanged with the activation of 5-HT1ARs by acute treatment with 8-OH-DPAT alone compared to the vehicle treatment. In addition, treatment with EB alone had no significant effect on the PIASxα expression level in comparison to vehicle-treated rats.

For PIAS3 (Figure 2C), there were no significant effects of the drug treatments. A two-way ANOVA analysis showed no significant effect of EB, 8-OH-DPAT treatment, or an interaction on the expression level of PIAS3 (two-way ANOVA, main effect of EB: F(1,8) = 0.025, *p* = 0.87; main effect of 8-OH-DPAT treatment: F(1,8) = 0.0061, *p* = 0.940; interaction between EB treatment and 8-OH-DPAT treatment: F(1,8) = 0.009, *p* = 0.92).

For PIASy, a two-way ANOVA showed a significant effect of EB treatment but not 8-OH-DPAT treatment or an interaction between EB and 8-OH-DPAT treatment (Figure 2D). Tukey’s multiple comparisons post hoc test shows the expression level of PIASy was significantly increased in the rats treated with EB alone, compared to vehicle-treated rats. However, no effect on PIASy expression was seen in the 8-OH-DPAT-treated group compared to vehicle-treated rats or the rats treated with EB and 8-OH-DPAT together. Intriguingly, there was no significant difference between EB-treated rats and rats co-treated with EB and 8-OH-DPAT (two-way ANOVA, main effect of EB treatment: F(1,8) = 8.463, *p* = 0.0196; main effect of 8-OH-DPAT treatment: F(1,8) = 0.152, *p* = 0.706; interaction between EB treatment and 8-OH-DPAT treatment: F(1,8) = 3.760, *p* = 0.088).

### 2.3. The Effects of the Overexpression of SENP Proteins on the SUMOylation of 5-HT1ARs

**Figure 3:**
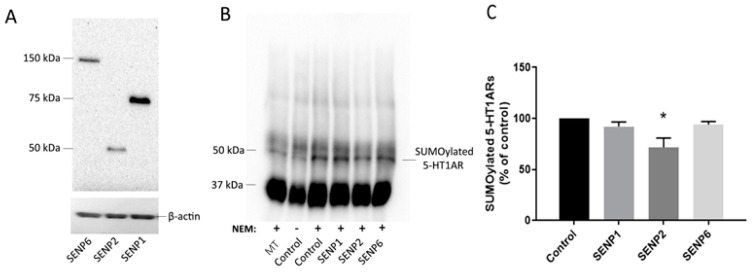
SENP2 deSUMOylated 5-HT1ARs. N2a cells were transfected with flag-tagged SENP plasmid constructs and were harvested 48 h after transfection. Immunoblot showing the expression of SENPs in the membrane fractions of N2a cell lysates using an anti-flag antibody (**A**). The membrane fraction of cells transfected with 5-HT1AR and SENP expression vectors were immunoprecipitated with SUMO-1 antibody and then examined on an immunoblot with a 5-HT1AR antibody to detect SUMOylated 5-HT1ARs (**B**). The cell lysates were prepared with or without the addition of NEM as indicated. Note that without NEM the SUMOylated 5-HT1AR band is less intense, verifying the protein band is a SUMOylated protein. Mock transfection (MT) is a control and indicates cells were treated with the transfection protocol and reagents but plasmid DNA was not included. The groups marked as controls were transfected with 5-HT1AR and SUMO-1 expressing plasmids. Graph showing the quantification of the SUMOylated 5-HT1ARs (**C**). Data are shown as mean ± SEM and the experiments were performed 4 independent times (*n* = 4). One-way ANOVA shows a significant effect of SENP transfection on SUMOylated 5-HT1AR levels (F(3,12) = 4.907, *p* < 0.0001). Dunnett’s multiple comparisons post-hoc test shows that SUMOylated 5-HT1AR significantly decreased in the group transfected with SENP2 as compared to the control, * indicates *p* < 0.05.

Flag-tagged SENP plasmid constructs were transfected into N2a cells to verify the expression of the appropriate size bands (Figure 3A). Since the protein levels of the transfected SENP proteins were similar to each other, no further analysis was performed as it was for the PAIS proteins. To determine which SENP enzyme is involved in the deSUMOylation of 5-HT1ARs, we overexpressed 5-HT1AR and SUMO1 along with SENP1, SENP2, SENP6, or an empty vector. Membrane fractions of the cell lysates were immunoprecipitated with a mouse anti-SUMO1 antibody and immunoblotted with a rabbit anti-5-HT1AR antibody (Figure 3B). Results showed that SENP2 significantly decreased the amount of SUMOylated 5-HT1AR as compared to the control when a one-way ANOVA was performed followed by Dunnett’s multiple comparisons test (F(3,12) = 4.907, *p* < 0.0001) (Figure 3C).

### 2.4. The Effects of Acute 8-OH-DPAT on the PIAS and SENP Proteins in the Rat Cortex

**Figure 4:**
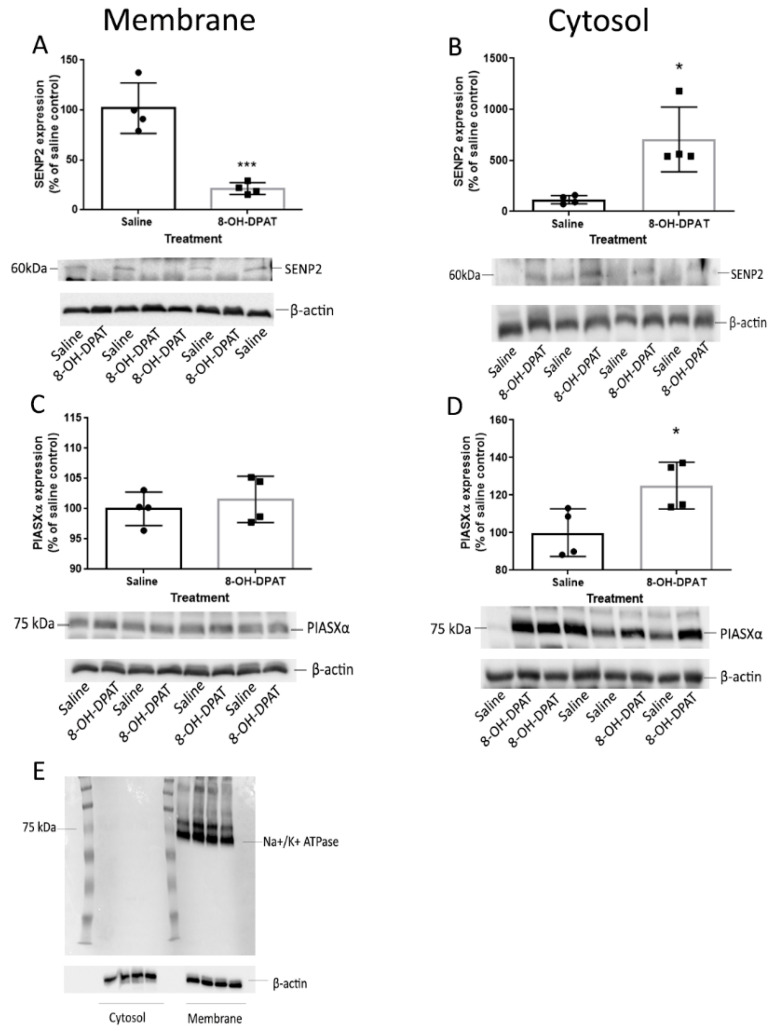
The effect of 8-OH-DPAT on PIASxα and SENP2 in rat frontal cortex. Membrane and cytosol fractions of frontal cortex from four rats treated with 8-OH-DPAT and four with saline were examined on immunoblots. Experiments were repeated three independent times. (**A**) In the membrane fraction of frontal cortex, SENP2 was significantly decreased in 8-OH-DPAT-treated rats compared to saline-treated controls (*** indicates unpaired *t*-test *p* = 0.0008). (**B**) SENP2 was significantly increased in the cytosol fraction from 8-OH-DPAT-treated compared to saline-treated rat frontal cortex (* indicates unpaired *t*-test *p* = 0.01). (**C**) There was no significant difference in PIASxα levels in the membrane fraction of the 8-OH-DPAT-treated compared to saline-treated rat frontal cortex. (**D**) There was, however, a small but significant increase in PIASxα levels in the cytosol fraction of frontal cortex from rats treated with 8-OH-DPAT compared to saline treated controls (* indicates unpaired *t*-test *p* = 0.03). (**E**) To verify separation of the membrane and cytosol fractions of the tissue samples, five samples of membrane and cytosol fractions were examined on an immunoblot with an antibody directed against the membrane protein Na^+^/K^+^ ATPase and protein bands were present only in the membrane fractions.

Since SENP2 is involved in the deSUMOylation of 5-HT1ARs, we hypothesized that SENP2 expression would decrease in the membrane fraction of the frontal cortex in rats treated with 8-OH-DPAT treatment, a treatment that leads to an increase in the SUMOylation of 5-HT1ARs [10]. To determine if the SENP2 expression decreases with 8-OH-DPAT treatment, SENP2 protein levels were examined on immunoblots. Membranes (Figure 4A) and cytosol fractions (Figure 4B) of the frontal cortex from rats treated with 8-OH-DPAT or saline were examined on immunoblots with an anti-SENP2 antibody. We observed one of the SENP2 isoforms at 60 kDa molecular weight. Using unpaired *t*-tests, there were significant differences in the levels of 60 kDa molecular weight isoforms; there was a 4.8-fold decrease in the membrane (*p* < 0.001) and a six-fold increase in the cytoplasmic fractions (*p* = 0.01) in the 8-OH-DPAT-treated group compared to the saline-treated group (Figure 4A,B). We also examined the levels of PIASxα in the membrane (Figure 4C) and cytosol (Figure 4D) fractions of the frontal cortex of rats treated with 8-OH-DPAT and saline. There was no significant difference in the membrane fraction (*p* = 0.54) and a small but significant (*p* < 0.03) increase in PIASxα in the cytoplasmic fraction. The separation of the membrane and cytosol fractions were verified using immunoblots for the membrane selective protein Na^+^/K^+^ ATPase (Figure 4E).

## 3. Discussion

Protein SUMOylation is an important post-translational modification that has recently gained attention. Although the vast majority of studies on SUMOylation focus on the regulation of nuclear proteins, studies have revealed that SUMOylation can also play an important role in regulating the function of extra-nuclear proteins [19]. In the last several years, a role for SUMOylation in neurodegenerative diseases, cerebral ischemia, and synaptic function has become apparent [20,21,22,23].

We previously reported that 5-HT1ARs are SUMOylated, and that agonist stimulation increases the SUMOylation of 5-HT1ARs in the frontal cortex and hypothalamus [10]. EB facilitates the effect of agonist stimulation of the receptor, resulting in significantly more SUMO1–5-HT1ARs than a treatment with agonist alone [10]. The purpose of the current studies is to elucidate the mechanisms regulating the SUMOylation of 5-HT1ARs. To this end, we first focused on the PIAS E3 ligases which facilitate SUMOylation and then SENP proteins which deSUMOylate proteins. Previous studies demonstrated that local concentrations of PIAS E3 ligases and SENPs can regulate the SUMOylation of target proteins [18,24], so we examined the levels of these enzymes in the membrane and cytosol fractions to determine if a change in the membrane levels is related to an increase in the amount of SUMOylated 5-HT1ARs, since SUMOylated 5-HT1ARs exist only in the membrane fractions [10]. Although previous studies reported PIAS and SENP proteins distributed primarily in the nucleus and cytosol of cells [11,25], our results show PIAS and SENP proteins distributed in the membrane fraction of N2a cells and the rat PVN and frontal cortex. Transfected PIASxα proteins were expressed at low levels in the whole cell lysate, but a higher proportion of PIASxα was expressed in the membrane fraction than in the whole cell lysate, suggesting PIASxα is positioned to participate in the SUMOylation of 5-HT1ARs which only exist in the membrane fraction [10]. Although there are low levels of endogenous PIAS proteins in the membrane fraction (Figure 1C), the increase in PIASxα in the membrane following transfection resulted in increased SUMO1–5-HT1ARs. Based on the transfection data, our interpretation is that PIASxα is the primary PIAS protein that catalyzes SUMO1 binding to 5-HT1AR and that low levels of endogenous PIASxα catalyze the SUMOylation of 5-HT1AR, as seen in the vector transfected cells. PIASxα selectively increases SUMO1–5-HT1ARs even though the expression level of transfected PIASxα is low compared to other transfected PIAS proteins. Despite much higher amounts of PIAS1 and PIASy proteins in the whole cell lysate preparation as well as the membrane fraction of N2a cells after transfection, high PIAS1 and PIASy did not increase SUMOylation of 5-HT1ARs over the levels that occur with the endogenous expression of PIAS proteins.

In this study, we found that PIASxα is increased in the membrane fraction of PVN in rats treated with EB and 8-OH-DPAT, suggesting that this increase in PIASxα is responsible for the increase in 5-HT1ARs SUMOylation induced by EB and 8-OH-DPAT [10]. It is important to note that the EB treatment was once daily for two days and then the agonist treatment was 5–15 min before sacrifice in the experiments examining PIASxα and those demonstrating increased 5-HT1AR SUMOylation. The two days of EB administration is the minimum treatment time for EB to reduce 5-HT1AR and hypothalamic pituitary adrenal (HPA) axis signaling, an important biomarker for depression [8]. Both 5-HT1ARs and estrogens are known to impact mood, especially in depression and anxiety. Numerous studies demonstrate that estrogens reduce 5-HT1AR signaling not only in the HPA axis [9,10] but also in the hypothalamus, hippocampus, dorsal raphe, and cortex [7,26,27]. In this study we focused on the frontal cortex and PVN due to the importance of these brain regions in depression and anxiety, as well as our previous experiments demonstrating the effects of 8-OH-DPAT and the combination of 8-OH-DPAT on the frontal cortex and hypothalamus, respectively. One of the mechanisms by which estrogens could reduce 5-HT1AR signaling is via SUMOylation, as our previous data suggests that the SUMOylation of 5-HT1ARs could reduce signaling via a reduced interaction with Gα proteins [10]. Mize et al. [27] also reported a reduction in G-protein coupling to 5-HT1ARs after EB treatment. The SUMOylation of proteins often alters interactions with their binding partners consistent with SUMOylation causing a change in receptor G-protein coupling. The effects of EB on 5-HT1AR SUMOylation and signaling may contribute to the increased incidence of mood disorders in women with fluctuations in estrogens such as during the perimenopausal period.

The administration of EB alone did not have significant effects on the levels of PIASxα, a finding consistent with our previous results, that the treatment of EB alone did not increase the SUMOylation of 5-HT1ARs. EB treatment alone did significantly increase the expression level of PIASy in the membrane fraction of the rat PVN. Since PIASy did not alter the SUMOylation of 5-HT1ARs, the change in PIASy levels may be involved in EB-induced SUMOylation of other proteins such as RGSz1 [28]. RGSz1 can be SUMOylated and there are numerous isoforms of SUMOylated RGSz1 at approximately 35 kDa, 45 kDa, 50 kDa, 90 kDa, and 135 kDa in the rat cortex; EB treatment increased the 135-kDa RGSz1 in the PVN membrane fraction [28].

We found that agonist treatment alone increased the levels of SUMO1–5-HT1ARs in the membrane fraction of the rat frontal cortex, similar to that previously reported for the rat hypothalamic membrane and the cortical detergent resistant membrane fractions [10]. After we identified SENP2 as capable of deSUMOylating 5-HT1ARs, we compared the levels of SENP2 in the 8-OH-DPAT-treated rat frontal cortex to saline-treated controls to determine if a decrease in SENP2 in the membrane could underlie the agonist-induced increase in the SUMOylation of 5-HT1ARs. Indeed, SENP2 decreased in the membrane fraction and increased in the cytosol fraction of the 8-OH-DPAT-treated rat frontal cortex, suggesting a translocation of SENP2 proteins following agonist treatment. These results further suggest that the translocation of SENP2 away from the membrane is responsible for the increase in the SUMOylation of 5-HT1ARs after agonist treatment. Similarly, a previous study demonstrated that SENP1 is also highly mobile and translocates to the synapse after the stimulation of metabotropic glutamate receptors (mGluR) [24]. PIASxα does not appear to be involved in regulating the increase in 5-HT1AR SUMOylation after agonist treatment, as 8-OH-DPAT did not increase the expression of PIASxα in the membrane fraction of the PVN.

The mechanisms involved in translocation of PIASxα and SENP2 have not been investigated but might involve post-translation modifications. PIASxα is subject to both SUMOylation and phosphorylation, and SUMOylation is known to alter the cellular location of proteins [14]. However, it is not known if these modifications play a role in regulating the translocation of PIASxα. Phosphorylation of SENP2 promotes its translocation from the nucleus to the cytosol [29] but it is not known if phosphorylation alters the localization of SENP2 to the cell membrane.

To date, only four other GPCRs, cannabinoid receptor 1 (CB1), M1 muscarinic acetylcholine receptors (M1Rs) and mGluR7 and mGluR8 have been identified as SUMOylated in mammals [30,31,32,33,34,35]. In contrast to 5-HT1ARs, agonist treatment decreases the SUMOylation of M1Rs, CB1, and mGluR7, however, no data are available regarding the effects of SUMOylation on the function of mGluR8. The deSUMOylation of mGluR7 increases the internalization of the receptors [30]. The SUMOylation of M1Rs increases ligand affinity and signaling of M1Rs, as well as endocytosis [35]. In drosophila, the GPCR smoothened is SUMOylated, resulting in the stabilization of the receptor [36] as well as promoting the translocation of the receptor to the cell surface [37]. Clearly, the effects of SUMOylation on receptor signaling are receptor specific.

## 4. Materials and Methods

### 4.1. Cell Culture Experiments

N2a cells obtained from ATCC were maintained in 50% Dulbecco’s Modified Eagle Medium (1× DMEM, high glucose, pyruvate, ThermoFisher Scientific, Waltham, MA, USA) and 50% OptiMEM (ThermoFisher Scientific) supplemented with 10% Fetal Bovine Serum (FBS) (Atlanta Biologicals, Flowery Branch, GA, USA) and 1% penicillin-streptomycin solution (Sigma Aldrich, St. Louis, MO, USA). N2a cells were plated in 10 cm dishes at a density of 2.2 × 10^6^ cells per dish. After 16–24 h, cells were transfected with the mammalian expression plasmid constructs using Lipofectamine 3000 (ThermoFisher Scientific). The cell culture media was changed 6 and 24 h after transfection. To allow for the maximal expression of 5-HT1ARs for the SENP transfection experiments, cells were transfected with 5-HT1ARs plasmids 16 h after transfecting SENP plasmids. Subsequently, cells were harvested at 48 h after SENP transfection and 32 h after 5-HT1AR transfection. In all other experiments, cells were harvested 48 h after transfection. Cells were washed with a phosphate-buffered saline and hypotonic buffer (0.25 M sucrose 50 mM Tris, pH 7.5, 5 mM EDTA, 100 mM NaCl, and 20 mM N-ethylmaleimide (NEM)). The cells were harvested with a hypotonic buffer containing 20 mM NEM, 1/100 dilution of phosphatase inhibitors (Sigma Aldrich), and protease inhibitors (Sigma Aldrich) and were centrifuged for 1 h. Then, the supernatant was removed as the cytosolic fraction and the pellet was resuspended in the solubilization buffer (20 mM Tris, pH 8, 1 mM EDTA, 100 mM NaCl, (1% sodium cholate, 20 mM NEM, 1/100 dilution of phosphatase inhibitors, and protease inhibitors were added immediately before use)). After sonication, the vials were shaken horizontally at 4 °C for 1 h. After this, vials were centrifuged at 25,000× *g* for 1 h. The supernatant was removed as the membrane fraction. The samples were aliquoted and stored in −80 °C. A BCA assay (ThermoFisher Scientific) was used to measure the protein concentration.

### 4.2. Plasmid Constructs

The flag-tagged PIAS and SENP plasmid constructs, his-tagged 5-HT1AR and his- and HA-tagged SUMO-1 constructs were from Addgene (Watertown, MA, USA). The (K)DYK tagged 5-HT1AR construct was from GenScript (Piscataway, NJ, USA). The QIAGEN (Germantown, MD, USA) plasmid MIDI prep kit was used to isolate the plasmids.

### 4.3. Treatment of Rats and Preparation of Tissue Fractions

The frontal cortex was used to examine the effects of acute treatment with the 5-HT1A/7R agonist, 8-OH-DPAT, since it was previously demonstrated that 8-OH-DPAT increases the SUMOylation of 5-HT1AR in this brain region [10]. 8-OH-DPAT was purchased from Tocris (Ellisville, MO, USA), was dissolved in 0.85% NaCl at a concentration of 0.2 mg/mL and was administered at a dose of 0.2 mg/kg s.c. Solutions were made fresh before injection. Sixteen ovariectomized female rats were injected with either saline or 8-OH-DPAT (200 µg/kg) subcutaneously and then decapitated 5 min later. Brains were removed and the frontal cortex was dissected, frozen on dry ice, and stored at −80 °C until use. Tissue was homogenized and membrane and cytosol fractions were prepared as previously described [10]. Four rats from each treatment group were examined on each Western blot and three to five Western blots were used for each antibody tested.

The paraventricular nucleus of the hypothalamus (PVN) of rats used in this study was from ovariectomized female rats used in a previously published study to determine the effects of EB on oxytocin and ACTH responses [28]. Our previous study [10] demonstrated that 8-OH-DPAT increased the SUMOylation of 5-HT1ARs in the hypothalamus and EB synergized this increase. EB from Sigma-Aldrich was first dissolved in 100% ethanol to a concentration of 25 μg/mL and then diluted to the final concentration with sesame oil. The EB solution and sesame oil were administered at 0.4 ml/kg (the EB dose was 10 μg/kg subcutaneous (s.c.)). 8-OH-DPAT was prepared and administered as described above. Both solutions were made fresh before the injection. Rats were given unilateral intra-PVN injections of GPR30-mis-Ads, a control vector that had no effect, as previously described [28]. Five days after the injection, rats were treated with either EB (10 μg/kg, 0.4 mL/kg, s.c.) or sesame oil once daily for 2 days. Twenty hours after the last injection, rats were treated with 8-OH-DPAT (200 μ/kg, s.c.) or saline and decapitated 15 min after the treatment. Brains were removed and the PVN was isolated and stored at −80 °C until use; the PVN was then homogenized and membrane and cytosol fractions were prepared as previously described [10]. Membrane fractions from three rats for each treatment group were used for immunoblots to examine the relative expression of PIAS proteins. All animal experiments were conducted according to the National Institutes of Health guide for the care and use of laboratory animals and was approved by the IACUC at the University of Kansas.

### 4.4. Immunoprecipitation

Immunoprecipitation was conducted using 250 µg to 450 µg of protein of the membrane fraction of N2a cells. The preparation of the membrane fraction is described above. The sample was added to 50 µL prewashed protein G agarose (Invitrogen ThermoFisher Scientific, Waltham, MA, USA) in a total volume of 250 µL IP buffer (50 mM Tris, pH 7.4, 10 mM EGTA, 100 mM NaCl, 0.5% Triton X-100, containing 20 mM NEM, 1× protease inhibitor cocktail, 1× phosphatase inhibitor cocktails I and II (Sigma Aldrich, St. Louis, MO, USA)) and rotated at 4 °C for 1 h. After centrifugation at 10,000 rpm at 4 °C for 10 min, the supernatant was incubated with a 2.5 μg anti-SUMO1 mouse antibody (Cat#sc-5308, Santa Cruz Biotechnology, Inc., Dallas, TX, USA) or mouse IgG control (Cat#sc-2025, Santa Cruz Biotechnology) on a rotator at 4 °C overnight. The solutions were added to 100 µL of pre-washed beads and rotated at 4 °C for 2 h. The protein G beads–immune complex was centrifuged at 1000 rpm for 3 min at 4 °C. The pellet was washed with a 0.5 ml ice-cold IP buffer 3 times. After washing, the proteins were eluted from the immune complexes using the 50 µL 2× sample buffer with β-mercaptoethanol and incubated at 95 °C for 5 min followed by centrifugation at 12,000 rpm for 5 min at room temperature. The eluate was stored in −80 °C or was loaded onto 10% SDS-PAGE gels.

### 4.5. Immunoblot Assays

Protein samples were resolved using 10% SDS-PAGE and then transferred to polyvinylidene fluoride (PVDF) membranes. After transferring, the PVDF membranes were incubated in 5% non-fat milk in Tris-buffered saline, pH 7.6, with 0.1% Tween-20. The membranes were incubated overnight with primary antibodies (PIAS antibodies (1:2000) from Dr. Yoshiaki Azuma, [38], rabbit anti-5-HT1AR (1:1000, Thermo Fisher Scientific Cat#PA5-28090), rat-anti-flag (1:2000 Agilent Technologies, (Santa Clara, CA, US), Cat#200474-21), rabbit anti-SENP2 (1:500, Invitrogen, Thermo Fisher Scientific, Cat# PA5-86255), mouse anti-Na^+^, K^+^ ATPase (1:1000, Millipore Sigma, (Burlington, MA, USA) Cat#05-369), and mouse-anti-β actin (1:20,000, MP Biomedicals, LLC, (Irvine, CA, USA) Cat#691001)). Immunodetection was performed using an ECL kit (Millipore Sigma or Bio-Rad Laboratories, Hercules, CA, USA) and ImageLab 3.0 software (Bio-Rad Laboratories). The intensity of bands was normalized to the amount of protein loaded in each lane, the mean intensity of the bands of the empty vector transfected samples, and the beta-actin band.

### 4.6. Statistical Analysis

All statistical analyses were conducted using GraphPad Prism version 9.1 (San Diego, CA, USA) software. The Shapiro–Wilk test for normality and the Brown–Forsythe–Levene test for homogeneity of variance were used to determine if the data met the requirements for a parametric analysis of variance. Immunoblot data were analyzed by a one-way or two-way analysis of variance (ANOVA) followed by post-hoc tests; Dunnett’s was used to compare only to the control group, or Tukey’s multiple comparisons test was used to identify differences among the treatment groups where appropriate. All data are represented as mean ± SEM.

## 5. Conclusions

In summary, this study demonstrated that SENP2 deSUMOylates 5-HT1ARs and the translocation of SENP2 away from the membrane to the cytosol is caused by an acute agonist treatment which correlates with the increase in SUMO1–5-HT1ARs in the cell membrane. In contrast, PIASxα increases SUMO1–5-HT1Ars, and treatment with an agonist and EB increases PIASxα in the membrane and increases SUMO1–5-HT1ARs. Interestingly, the combination of EB and 8-OH-DPAT causes an increase in PIASxα in the membrane and increases the SUMOylation of 5-HT1Ars, although EB alone causes neither. These consistent relationships suggest that there are two different mechanisms by which EB and 8-OH-DPAT induce the increase in the SUMOylation of 5-HT1ARs. Targeting PIASxα and SENP2 to alter the SUMOylation of 5-HT1ARs could have important clinical relevance for the therapeutic intervention for anxiety disorders, as well as other neuropsychiatric disorders in which 5-HT1ARs are implicated.

## Data Availability

Data is contained within this article.
